# Catch-up immunization for adolescents and young adults during pre-travel consultation in Japan

**DOI:** 10.1371/journal.pone.0258357

**Published:** 2021-10-14

**Authors:** Kei Yamamoto, Michiyo Suzuki, Mugen Ujiie, Shuzo Kanagawa, Norio Ohmagari

**Affiliations:** 1 Disease Control and Prevention Center, National Center for Global Health and Medicine, Shinjuku-ku, Tokyo, Japan; 2 MARU, Tokyo Business Clinic, Chiyoda-ku, Tokyo, Japan; Rutgers New Jersey Medical School, UNITED STATES

## Abstract

Rubella and measles outbreaks in adults occur because of unimmunized or partially immunized status. Travel clinics play an important role in catch-up measles, rubella, mumps, and varicella immunization for adults. We evaluated the need for catch-up measles, rubella, mumps, and varicella immunization by young adults at our travel clinic. This retrospective observational study was conducted at the National Center for Global Health and Medicine from June 1, 2017 to May 31, 2018. Adults aged 16–49 years who received pre-travel consultation and had childhood immunization records were included. Individuals who fully or partially received planned measles, rubella, mumps, and varicella catch-up immunization were classified as “immunized.” We calculated the proportion of “immunized” individuals and analyzed the factors associated with catch-up measles, rubella, mumps, and varicella immunization at pre-travel consultation using logistic regression analysis. Overall, 3,456 individuals received pre-travel consultations during the study period; 827 (336 men, median age 22 years) had childhood immunization records. The most common trip purposes were study (33%) and tourism (24%). The most common destination was Asia (39%). Catch-up immunization of any measles, rubella, mumps, and varicella vaccine was needed by 755 individuals. After consultation, 20–46% of these participants who needed catchup immunization received at least one dose of immunization. Factors that are negatively associated with measles, rubella, mumps, and varicella catch-up immunization were tourism (odds ratio 0.37 to 0.58), yellow fever vaccination (0.45 to 0.50) (excluding varicella), and each disease history (0.13 to 0.40) (excluding rubella and varicella). Further studies are needed to identify barriers to catch-up immunization.

## Introduction

**Fig 1:**
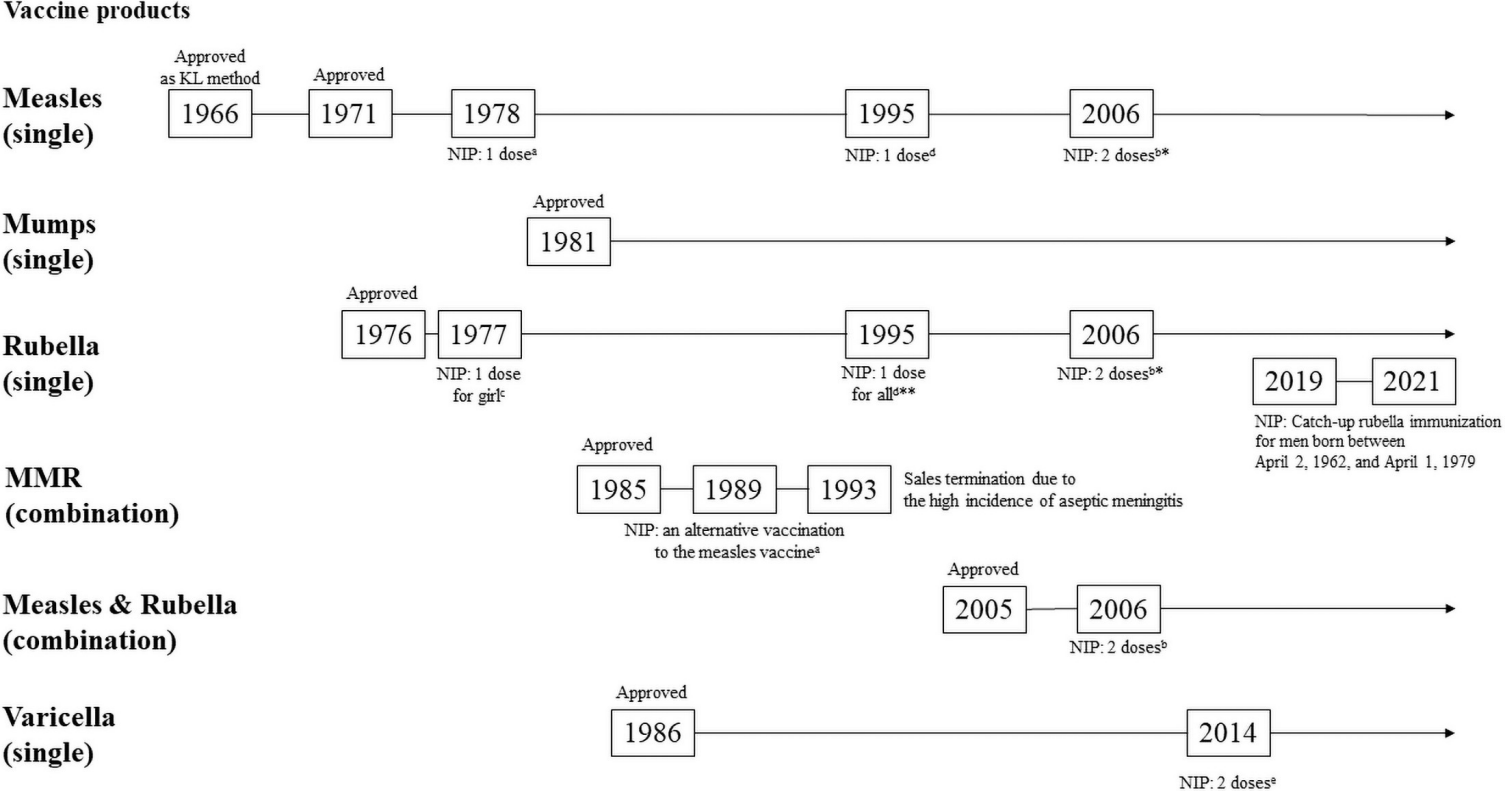
History of approval and the national immunization program guidelines for measles, rubella, mumps, and varicella vaccine. *2008–2012 Catch-up second doses of immunization for 13- and 18-year-old individuals. **1995–2003 Catch-up immunization for junior high school-aged boys and girls. ^a^ For infant and children aged on 1 to 6 years. ^b^ For infant aged 1 year (1st dose) and children aged 5 to 6 years (2nd dose). ^c^ For children in 2nd grade of middle high school (aged 13 to 14 years). ^d^ For infants and children aged 1 to 7.5 years. ^e^ For infants and children aged 1 to 3 years (2 doses, at least 3 months interval). KL method: Combined use of inactivated (killed) and live measles vaccines. NIP, National immunization program; MMR, measles, mumps, rubella vaccine.

Although measles and rubella are vaccine-preventable diseases, their outbreaks, especially in adults, occur because of unimmunized or partially immunized individuals [1–3]. In Japan, measles and rubella cases occur mainly among young adults in their 20s-40s [2]. Two doses of combined measles and rubella vaccine have been administered to children at a relatively high vaccination rate, as shown in the history of the National Immunization Program in Japan (Fig 1). However, Japanese adult men were not immunized with two doses of measles vaccine and rubella vaccination in the national program. The government carried out a catch-up immunization program for junior high school-aged boys and girls from 1995 to 2003 [4] for two different measles and rubella immunization doses. Catch-up immunization of measles and rubella combined vaccine for 13- and 18-year-old individuals were done between 2008 and 2012. The vaccination rate for the former was relatively high at around 90%, while the rate for the latter was <80%. To address this, the Japanese Ministry of Health, Labor, and Welfare provided a means for males born between April 2, 1962, and April 1, 1979 (a generation that was not vaccinated against rubella) to test for antibodies and to receive subsidized rubella or measles and rubella vaccines, in December 2018 [5]. In the first six months of the program, only 16% of the target population received the antibody test [6].

Adults only consider the need for vaccinations on few occasions, such as when they are thinking about getting pregnant, when they have immunosuppressive or infectious diseases, or when vaccines are needed for occupational reasons (such as in the medical profession). The pre-travel consultation is one of such occasions, and travel clinics play an important role in catch-up MMRV immunization for adults [7,8]. In Japan, the number of Japanese leaving the country exceeded 20 million in 2019, and 11 million (59.0%) of them were adolescents and adults aged 15–49 [9]. Many of the clients attending pretravel consultations are young adults [10]. No previous reports exist on catch-up vaccination among Japanese who traveled abroad. In the United States, among attendees aged ≥18 years who presented to the Global TravEpiNet (GTEN)-registered facilities for pre-travel consultations, about 47% received measles-containing vaccines [11]. However, there are differences by countries in the cost-subsidy system and history of introducing the national immunization program. We evaluated the need for and implementation of catch-up MMRV immunization by adolescents and adults aged 16–49 years at our travel clinic in Japan.

## Material and methods

This retrospective observational study was conducted at the National Center for Global Health and Medicine hospital (NCGM), where approximately 3,000 individuals received pre-travel consultations annually. At NCGM, eight physicians and four fellows oversaw pre-travel consultations. Although there was no specific guideline for MMRV vaccination, the hospital actively recommended two doses of MMRV vaccines for catch-up immunization during the travel clinic attendees’ lifetime.

Individuals aged 16–49 years who received pre-travel consultation and had their childhood immunization records between June 1, 2017 and May 31, 2018 were included in this study. We (MS and KY) accessed the patients’ medical records at the hospital between October 4, 2018, and December 31, 2018, and created a dataset in Microsoft Excel 2019 (Microsoft Corporation, Redmond, WA, USA). Information about the study, which advised that patient records might be used in medical research, was shown on both the hospital website and bulletin board with an opt-out participation option. This was substituted for the participants’ consent. The protocol of this study including the opt-out consent method was approved by the ethical committee of the National Center for Global Health and Medicine (NCGM-G-002551-00) on 9 July 2018.

Information on age, sex, past and present medical history (self-reported or documented), destination, the purpose of travel, departure date, immunization history, MMRV antibody results, and scheduled vaccines after consultation was extracted from the participants’ medical records at the travel clinic. Current illness and medical history were obtained through an interview during pretravel consultation to identify any underlying disease that would be a contraindication to live-attenuated vaccines. Contraindications to MMRV were immunocompromised host, use of immunosuppressive drugs, pregnancy, and anaphylaxis to the same vaccine in the past. Immunocompromised host was defined as receiving chemotherapy or radiation therapy for malignancy, congenital immunodeficiency, or HIV infection with a CD4 positive lymphocyte count <200 /μL. Patients taking immunosuppressive drugs were contraindicated in principle, but those on low-dose corticosteroids, 6-mercaptopurine, azathioprine, and methotrexate, used at the discretion of the physician based on Infectious Diseases Society of America (IDSA) guidelines, were included [12].

Following the United Nations geographic classification [13], we classified 209 countries and regions into “Asia,” “Oceania and Micronesia,” “North America,” “Central and South America,” “Europe,” “Middle East,” “Africa,” and others. “Asia” included East Asia, Southeast Asia, and South Asia, and West Asia was classified as “the Middle East.” “Central and South America” includes the following regions: Central America, South America, and the Caribbean. Australia and New Zealand, Micronesia, Polynesia, and Melanesia were classified as “Oceania and Micronesia.” Travel across two or more regions, excluding transit, was defined as travel to multiple regions. The purpose of travel was tourism (packaged and non-packaged tours), business, accompanying family members, study, volunteering, visiting friends and relatives (VFR), etc. A school trip was defined as “study,” whereas study tour outside of school was defined as “volunteering.” If there were more than one purpose of travel, non-tourism purposes were categorized as the priority. If it was impossible to determine priority of travel, the travel purposes were categorized as “Others.” The time to departure was defined as the interval from the initial visit to our travel clinic to departure, which was categorized as follows: within 1, 2, 4,>4 weeks, while undetermined ones were categorized as unknown.

We defined the need for catch-up MMRV immunization as fewer than two doses of each MMRV vaccine and no proven immunity (based on MMRV antibody tests or past medical history of chickenpox/shingles). Individuals who received planned full or partial MMRV catch-up immunization were defined as “immunized”.

### Statistical analysis

Discrete data and continuous data were expressed as numbers (percentages) and medians (interquartile ranges), respectively. We calculated the proportion of “immunized” individuals after pre-travel consultation. Logistic regression analysis (stepwise) was used to determine the factors associated with catch-up MMRV immunization at pre-travel consultation. The factors included were age, sex, travel to North America, purpose (tourism, study, or business), first clinic visit and departure interval, planning for yellow fever immunization, and history of the disease (excluding varicella immunization). IBM SPSS Statistics software for Windows, version 26.0 (IBM Corp., Armonk, NY, USA) was used for all analyses. The figures were created in Microsoft Excel 2019 and Microsoft PowerPoint 2019 (Microsoft Corporation, Redmond, WA, USA).

## Results

### Characteristics of the subject

**Fig 2:**
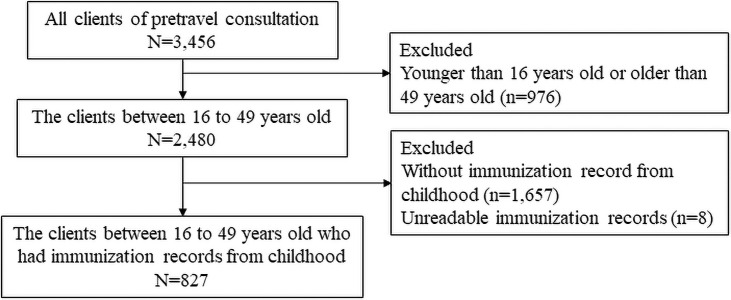
Study flow diagram. Number of eligible patients among those who visited the hospital between June 1, 2017, and May 31, 2018.

**Fig 3:**
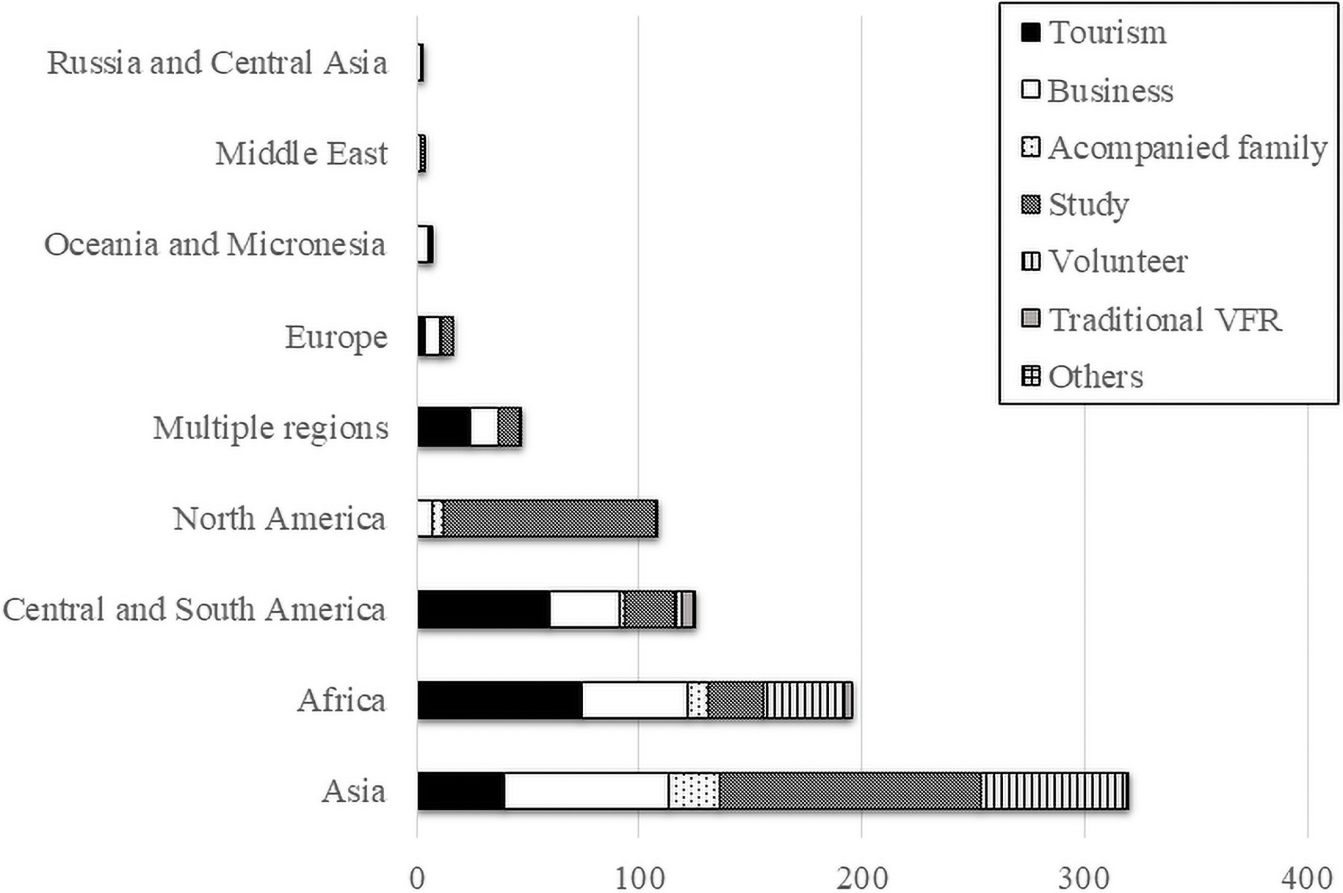
Travel destinations and purpose. The purpose of travel was considered to be one per participant. Those traveling to more than one region were categorized as “Multiple regions” and were not included in the total number for each region. Participants who traveled to their own or their parents’ country of origin were classified as “Traditional VFR”. VFR; visiting friends and relatives.

A total of 827 individuals (336 men) were included (Fig 2). The median age was 22 (20–29) years. The median interval from the first clinic visit to departure was 45 days (25–71 days). The most common destination was Asia (39%), and the most common trip purposes were study (33%) and tourism (24%) (Fig 3). Medical histories of measles, rubella, mumps, and varicella were present in 29 (4%), 32 (4%), 163 (20%), and 370 (45%) participants, respectively. Serological testing was performed on 86 participants (10%), including 44, 50, 66, and 66 for measles, rubella, mumps, and varicella, respectively. Seropositive rates among those that underwent serological testing were 97.7%, 71.2%, 88.0%, and 93.9% for measles, rubella, mumps, and varicella, respectively (S1 Table). The characteristics of the travelers by age group are shown in Table 1.

**Table 1:** Characteristics of the participants.

Age group	All	16–19 years	20–29 years	30–39 years	40–49 years
N	**827**	177	448	154	48
Males, n	**336 (40.6%)**	53 (29.9%)	191 (42.6%)	68 (44.2%)	24 (50.0%)
Age, median years (IQR)	**22 (20–29)**	19 (18–19)	22 (20–25)	34 (31–36)	44 (41–46)
Duration to departure, n					
within 1 week	**27 (3.3%)**	4 (2.3%)	14 (3.1%)	6 (3.9%)	3 (6.3%)
1 to 2 weeks	**68 (8.2%)**	9 (5.1%)	42 (9.4%)	16 (10.4%)	1 (2.1%)
2 to 4 weeks	**159 (19.2%)**	32 (18.1%)	89 (19.9%)	28 (18.2%)	10 (20.8%)
over 4 weeks	**558 (67.5%)**	131 (74.0%)	299 (66.7%)	98 (63.6%)	30 (62.5%)
Unknown	**15 (1.8%)**	1 (0.6%)	4 (0.9%)	6 (3.9%)	4 (8.3%)
Purpose of travel, n					
Tourism	**201 (24.3%)**	22 (12.4%)	118 (26.3%)	44 (28.6%)	17 (35.4%)
Business	**189 (22.9%)**	2 (1.1%)	98 (21.9%)	70 (45.5%)	19 (39.6%)
Accompaniment	**42 (5.1%)**	1 (0.6%)	7 (1.6%)	24 (15.6%)	10 (20.8%)
Study	**277 (33.5%)**	95 (53.7%)	170 (37.9%)	11 (7.1%)	1 (2.1%)
Volunteering	**106 (12.8%)**	52 (29.4%)	51 (11.4%)	2 (1.3%)	1 (2.1%)
Traditional VFR	**10 (1.2%)**	4 (2.3%)	4 (0.9%)	2 (1.3%)	0
Others	**2 (0.2%)**	1 (0.6%)	0	1 (0.6%)	0
Destination, n					
Asia	**320 (38.7%)**	93 (52.5%)	156 (34.8%)	50 (32.5%)	21 (43.8%)
Oceania and Micronesia	**8 (1.0%)**	1 (0.6%)	5 (1.1%)	1 (0.6%)	1 (2.1%)
North America	**108 (13.1%)**	37 (20.9%)	58 (12.9%)	12 (7.8%)	1 (2.1%)
Central and South America	**125 (15.1%)**	8 (4.5%)	79 (17.6%)	30 (19.5%)	8 (16.7%)
Europe	**16 (1.9%)**	3 (1.7%)	3 (0.7%)	9 (5.8%)	1 (2.1%)
Middle East	**4 (0.5%)**	1 (0.6%)	3 (0.7%)	0	0
Africa	**196 (23.7%)**	33 (18.6%)	106 (23.7%)	43 (27.9%)	14 (29.2%)
Russia and Central Asia	**3 (0.4%)**	0	3 (0.7%)	0	0
Others/not determinate	**1 (0.1%)**	0	1 (0.2%)	0	0
Multiple regions	**46 (5.6%)**	1 (0.6%)	34 (7.6%)	9 (5.8%)	2 (4.2%)
Past medical history of MMRV, n					
Measles	**29 (3.5%)**	2 (1.1%)	14 (3.1%)	7 (4.5%)	6 (12.5%)
Rubella	**32 (3.9%)**	0	13 (2.9%)	15 (9.7%)	4 (8.3%)
Mumps	**163 (19.7%)**	47 (26.6%)	87 (19.4%)	18 (11.7%)	11 (22.9%)
Varicella	**370 (44.7%)**	85 (48.0%)	210 (46.9%)	58 (37.7%)	17 (35.4%)
Past immunization of MMRV vaccine, n					
No measles vaccination	**50 (6.0%)**	2 (0.2%)	11 (1.3%)	15 (1.8%)	22 (2.7%)
Measles 1 dose	**245 (29.6%)**	25 (14.1%)	82 (18.3%)	119 (77.3%)	19 (39.6%)
Measles 2 doses or more	**532 (64.3%)**	150 (84.7%)	355 (79.2%)	20 (13.0%)	7 (14.6%)
No rubella vaccination	**155 (18.7%)**	3 (1.7%)	34 (7.6%)	79 (51.3%)	39 (81.3%)
Rubella 1 dose	**202 (24.4%)**	27 (15.3%)	110 (24.6%)	60 (39.0%)	5 (10.4%)
Rubella 2 doses or more	**470 (56.8%)**	147 (83.1%)	304 (67.9%)	15 (9.7%)	4 (8.3%)
No mumps vaccination	**397 (48.0%)**	81 (45.8%)	199 (44.4%)	78 (50.6%)	39 (81.3%)
Mumps 1 dose	**367 (44.3%)**	80 (45.2%)	213 (47.5%)	67 (42.9%)	7 (14.6%)
Mumps 2 doses or more	**63 (7.6%)**	16 (9.0%)	36 (8.0%)	9 (5.8%)	2 (4.1%)
No varicella vaccination	**560 (67.7%)**	100 (56.5%)	287 (64.1%)	126 (81.8%)	47 (97.9%)
Varicella 1 dose	**246 (29.6%)**	69 (39.0%)	150 (33.5%)	27 (16.9%)	0
Varicella 2 doses or more	**21 (2.5%)**	8 (4.5%)	11 (2.5%)	1 (0.6%)	1 (2.1%)

IQR, interquartile range; VFR, visiting friends and relatives; MMRV, measles, rubella, mumps, varicella.

### The need for catch-up vaccinations and acceptance

**Fig 4:**
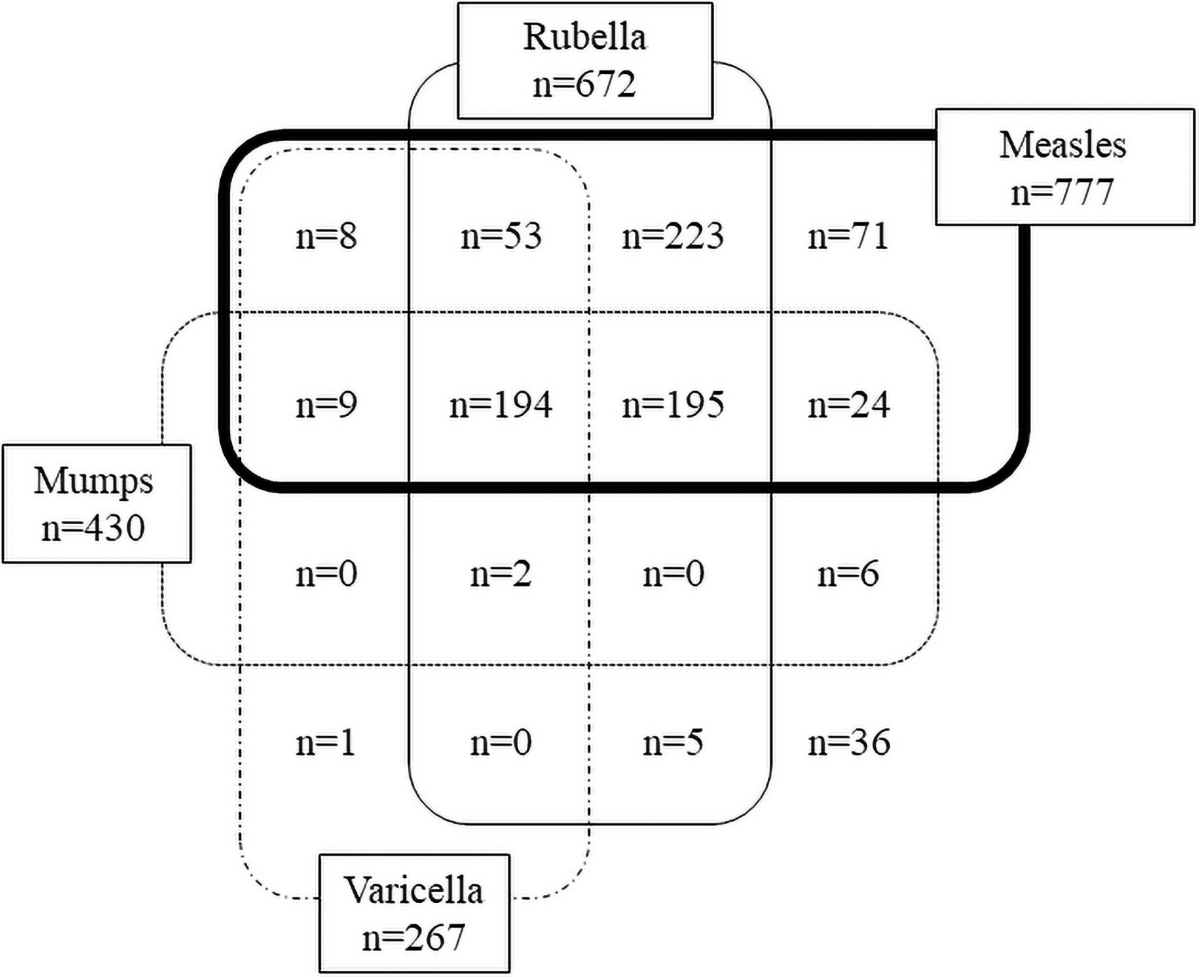
Details of MMRV vaccination status according to the immunization records. Vaccine records brought by patients were consulted, and subjects who had received at least one dose of measles, mumps, rubella, and varicella vaccine in each were counted.

The rate of receiving two doses of measles and rubella vaccines before pretravel consultation was highest in those aged 16–19 and 20–29 years, while receiving two doses of mumps or chickenpox vaccine was less than 10% in all other age groups (Table 1). Fig 4 shows the categorization of the MMRV vaccination history based on the vaccination records. Most of the participants had a history of measles and rubella vaccination, and fewer had been vaccinated against mumps and varicella. Of these, although 194 participants had been vaccinated against all of the MMRVs, the catch-up of two doses and catch-up immunization for any MMRV vaccine component were needed in 755 participants (91%) (Table 2). Mumps prevention required more vaccines than other diseases, with 192 cases requiring mumps vaccine alone and 525 cases requiring it in combination with other vaccines. This accounts for 95% of the participants that required catch-up vaccination. Measles was the least infectious disease that the participants required two doses of vaccine (5%) for, while other vaccines accounted for 17–43%. Participants were immunized with at least one dose in about 24–47% of all vaccines, but immunization rate of two doses was lower for all vaccines (10–33%) than for one dose (34–49%) (Table 2).

**Table 2:** Details of individuals requiring catch-up immunization.

N (1 dose needed/2 doses needed)	Total	Measles	Rubella	Mumps	Varicella
Measles only	**2**	2/0	-	-	-
Rubella only	**3**	-	1/2	-	-
Mumps only	**192**	-	-	100/92	-
Varicella only	**28**	-	-	-	21/7
Measles, rubella	**3**	2/1	2/1	-	-
Measles, mumps	**11**	9/2	-	6/5	-
Measles, varicella	**1**	1/0	-	-	1/0
Rubella, mumps	**36**	-	28/8	8/28	-
Rubella, varicella	**1**	-	1/0	-	1/0
Mumps, varicella	**193**	-	-	120/73	128/65
Measles, rubella, mumps	**106**	92/14	56/50	50/56	-
Measles, rubella, varicella	**0**	0/0	0/0	-	0/0
Measles, mumps, varicella	**3**	3/0	-	2/1	1/2
Rubella, mumps, varicella	**34**	-	29/5	15/19	14/20
All	**142**	118/24	70/72	57/85	35/107
Needed catch-up immunization (%)	**755 (91)**	227 (27)/ 41 (5)	187 (23)/ 138 (17)	358 (43)/ 359 (43)	201 (24)/ 201 (24)
The proportion of catch-up immunization after consultation (%)	1 completed dose needed	112/227 (49)	80/187 (43)	138/358 (39)	69/201 (34)
	2 completed doses needed	8/41 (20)	26/138 (19)	118/359 (33)	19/201 (10)
	At least 1 dose vaccination	123/269 (46)	135/325 (42)	296/718 (41)	96/402 (24)

The denominator for “Needed catch-up immunization” is the total number of participants included in the study, while for “The proportion of catch-up immunization after consultation,” it is the number of participants who needed catch-up vaccination.

### Factors associated with the implementation of catch-up vaccination

Factors associated with catch-up vaccine implementation for each MMRV vaccine revealed a lower likelihood of implementation of all vaccines among tourists (Table 3). The proportions of tourists, who required catch-up vaccination and received at least one dose measles, mumps, rubella, and varicella vaccines, after pretravel consultation, were low at 28.4%, 20.5%, 26.0%, and 11.6%, respectively. Yellow fever vaccination was associated with a lower likelihood of implementation of measles (odds ratio [OR]: 0.47, 95% confidence interval [CI]: 0.28–0.81), rubella (OR: 0.45, 95% CI: 0.26–0.74), and mumps (OR: 0.50, 95% CI: 0.34–0.75) vaccinations. The proportions of yellow fever vaccine recipients, who required catch-up vaccination and received at least one dose of measles, mumps, and rubella vaccines after pretravel consultation, were low at 33.3%, 20.5%, and 28.2%, respectively. Disease history of measles and mumps was associated with a lower likelihood of vaccine implementation, significantly (Table 3 and S1 Table). Compared with travelers to other destinations, travelers to North America were more likely to receive mumps (OR: 6.01, 95% CI: 3.51–10.38) and varicella (OR: 2.30, 95% CI: 1.20–4.39) vaccinations.

**Table 3:** Multivariate analysis of factors for implementing catch-up measles, mumps, rubella, and varicella immunization.

Odds ratio (95% confidence interval)	Measles	Rubella	Mumps	Varicella
N	268	325	717	402
Age (per 1 year older)	-	-	1.06 (1.03–1.08)	-
Sex (male)	-	-	-	-
North America	-	-	6.01 (3.51–10.38)	2.30 (1.20–4.39)
Tourism	0.37 (0.21–0.66)	0.44 (0.26–0.76)	0.58 (0.37–0.91)	0.37 (0.20–0.70)
Business	-	-	-	-
Study	-	-	-	-
Yellow fever immunization	0.47 (0.28–0.81)	0.45 (0.26–0.74)	0.50 (0.34–0.75)	-
History of the disease	0.13 (0.04–0.48)	0.40 (0.16–1.00)	0.27 (0.16–0.45)	Not applicable

Multivariate analysis using stepwise method was performed for participants who required catch-up vaccination for measles, rubella, mumps, and varicella.

The dependent variable was whether or not the corresponding vaccine was given for measles, rubella, mumps, and varicella.

The independent variables were age (per year), gender, travel to North America, travel for educational purposes, travel for work, travel for tourism, yellow fever vaccination, and history of each disease (except varicella).

## Discussion

In the present study, 94% of the participants needed some MMRV catch-up immunization. Catch-up mumps immunization was needed in most of these young adults, which had not been included in the routine national immunization program in Japan. In addition, varicella was not a routine vaccine in Japan during the study participants’ childhood. However, based on previous reports, participants with varicella history (about 45% of the participants in this study) were not vaccinated [14,15]. Almost half of the participants received the measles catch-up vaccine, including those requiring two doses of vaccine. Measles acceptance is consistent with a previous report from the US [9], where 47% of measles vaccine-eligible participants, who planned to go abroad, accepted measles, mumps, and rubella (MMR) vaccination. Although tourism is the most common travel purpose in patients with travel-related measles [16], 71.6% of tourists, in whom catch-up vaccinations were needed, did not receive measles vaccination in this study. All travelers need to know that MMRV is a highly contagious disease [17] and that prevention is essential, even for short-term travel, to reduce the spread of the disease across the countries [18].

In this study, subjects with a history of measles and mumps tended not to be immunized. Although some people had measles or mumps history, their immune status was not confirmed by serological test [19,20]. In practice, measles’s clinical diagnosis is often challenging, especially in secondary measles vaccine failure [21]. Mumps is also known to be less associated with sporadic cases of parotitis [22–24]. Therefore, although recall bias may be involved because the medical history in these reports was self-reported, MMR history is less credible than that of varicella, and we believe that aggressive catch-up immunization should be conducted for subjects with an MMR history. However, participants with measles and rubella medical history tend not to receive catch-up immunization. Although the reliability of history with varicella vaccination is generally higher than that of MMR, it has been suggested that vaccine failure due to varicella vaccination may be more difficult to judge by history [25]. As mentioned in Fig 1, since the varicella vaccine has only been introduced recently in Japan, we believe that varicella immunization history was somewhat reliable in this study.

Participants concurrently administered yellow fever vaccine had a low catch-up immunization rate for MMR. Physicians may avoid administering the MMR vaccine with yellow fever vaccine due to low immunogenicity reports with the co-administration in infants [26]. However, other than this previous literature, other studies reported that immune interference with the yellow fever vaccine did not occur with the measles-containing vaccines, including combined measles-rubella vaccines [27,28]. It is not clear to what extent these previous results could be applied to the present study because Silva et al. [26] reported that their study participants were 1-year old children and were tested in only one country. Further studies that will determine whether concurrent MMR vaccine and yellow fever vaccination in adults cause immune interference are needed.

In this study, only 10% of clients had MMRV serologic test results. Rapose reported that only 3% of patients who underwent the test were seronegative for measles [7]. Similarly, because seroprevalence data in Japan showed that only 2% of individuals aged 16–49 years were seronegative [29], testing may reduce the number of MMRV-eligible persons. However, for measles, a high antibody titer is required to prevent the disease [30]. Therefore, aiming for a high antibody titer may lead to inoculation after all, even if the antibody is measured. Medical practitioners also reported that about 20% of measles antibody titers are negative [31], and it is known that nearly 40–60% are not achieved when high standards were sought [32]. Although, if an individual has a vaccination record, it may be more cost-effective to give a total of two doses of vaccine than to test for antibody titers; further cost analysis and benefits of serological testing for MMRV in travel clinics are needed.

### Limitations

Unlike the data from GTEN [9], we do not know if participants accepted the vaccination or if the health care provider did not recommend vaccination in this case. Although we did not include the vaccines which the participants wanted to receive in our multivariate analysis, we would expect a higher propensity for vaccination if the vaccines which the participants wanted to receive included MMRV vaccines. In fact, catch-up vaccination for mumps and varicella was well accepted among travelers to North America in our study. Of these, 88% of participants visited pretravel consultation for the purpose of studying abroad (Fig 3). With most of the participants in this study, catch-up vaccines were well received because they are generally required to receive two MMRV vaccine doses in North American schools. The fact that catchup immunization rate was not related to the participants’ intention to study in other countries where MMRV vaccines are often not required, suggests an effect of the requirement of vaccine in this study (Table 3). However, although a multivariate analysis was performed excluding travelers to North America, the factors for catch-up immunization did not change significantly (S2 Table).

The study was also unable to document whether the cost of vaccination was borne by the individual, the company, or other organizations. In countries where vaccines are available freely, such as the UK, the cost was not cited as a reason to avoid vaccination [33], but in Japan, people have to pay for all the vaccines except for those in the national program. The cost per vaccine is approximately 6,000 to 20,000 JPY (60–200 USD); therefore, vaccination rate may be low among tourists without subsidy. However, immunization rate of MMRV was not high, even with business trips where companies often subsidize vaccination costs. It was impossible to conclude whether cost is a major barrier for catch-up immunization.

## Conclusions

Only about half of the travelers received catch-up MMRV immunization in pre-travel consultations in Japan. Many participants who planned to go on tourism, who received a yellow fever vaccine, or who had a history of each MMR did not receive catch-up immunization. It would be useful to identify the barriers to catch-up vaccination among adults for pre-travel MMRV consultations, which can be a risk even in short-term travels.

## Supporting information

S1 Table Vaccination rate on participants with past medical history or seropositive status of measles, mumps, rubella, or varicella. (DOCX)

S2 Table Multivariate analysis of factors for conducting catch-up measles, mumps, rubella, and varicella immunization excluding travelers to North America. (DOCX)

S1 Dataset (XLSX)
