# Supplementary material for: Catch-up immunization for adolescents and young adults during pre-travel consultation in Japan
Source: PLoS One. 2021 Oct 14;16(10):e0258357. doi: 10.1371/journal.pone.0258357 (PMC8516256; doi:10.1371/journal.pone.0258357)
Supplement: S1 Table — (DOCX) [file pone.0258357.s001.docx]

|  | Diseases | N | Vaccination rates (%)^a^ |
| --- | --- | --- | --- |
| Past medical history | Measles | 29 | 10.3% (3/29) |
|  | Mumps | 163 | 12.3% (20/163) |
|  | Rubella | 32 | 21.9% (7/32) |
|  | Varicella | 370 | 1.1% (4/370) |
| Seropositive | Measles | 43 | 9.3% (4/43) |
|  | Mumps | 47 | 2.1% (1/47) |
|  | Rubella | 44 | 11.4% (5/44) |
|  | Varicella | 62 | 0% (0/62) |
| ^a^The number of participants who received vaccination per disease at least one dose divided by the N) | | | |

Table S1. Vaccination rate on participants with past medical history or seropositive status of measles, mumps, rubella, or varicella.
