# Supplementary material for: Catch-up immunization for adolescents and young adults during pre-travel consultation in Japan
Source: PLoS One. 2021 Oct 14;16(10):e0258357. doi: 10.1371/journal.pone.0258357 (PMC8516256; doi:10.1371/journal.pone.0258357)
Supplement: S2 Table — (DOCX) [file pone.0258357.s002.docx]

| **Table S2. Multivariate analysis of factors for conducting catch-up measles, mumps, rubella, and varicella immunization excluding travelers to North America** | | | | |
| --- | --- | --- | --- | --- |
| Odds ratio  (95% confidence interval) | Measles | Rubella | Mumps | Varicella |
| N | 243 | 293 | 628 | 356 |
| Age (per year) | ‐ | - | 1.06 (1.03–1.09) | ‐ |
| Sex (male) | ‐ | ‐ | ‐ | ‐ |
| Tourism | 0.38 (0.21–0.68) | 0.48 (0.28– 0.82) | 0.60 (0.39–0.94) | 0.39 (0.20–0.74) |
| Business | ‐ | ‐ | ‐ | ‐ |
| Study | ‐ | ‐ | ‐ | ‐ |
| Yellow fever immunization | 0.53 (0.30–0.91) | 0.52 (0.31– 0.85) | 0.57 (0.38–0.84) | ‐ |
| History of the disease | 0.10 (0.02–0.48) | - | 0.30 (0.17-0.53) | Not applicable |
| Multivariate analysis using the stepwise method was performed on participants who required catch-up vaccinations for measles, rubella, mumps, and varicella.  The dependent variable was whether the corresponding vaccine was given for measles, rubella, mumps, and varicella or not.  The independent variables were age (per year), gender, travel for educational purposes, travel for work, travel for tourism, yellow fever vaccination, and history of each disease (except varicella). | | | | |
